# Full-Genome Sequence Analysis of a Variant Strain of Porcine Epidemic Diarrhea Virus in South Korea

**DOI:** 10.1128/genomeA.01116-14

**Published:** 2014-11-20

**Authors:** Sunhee Lee, Gun-Seok Park, Jae-Ho Shin, Changhee Lee

**Affiliations:** a Animal Virology Laboratory, School of Life Sciences, BK21 Plus KNU Creative BioResearch Group, Kyungpook National University, Daegu, Republic of Korea; b School of Applied Biosciences, College of Agriculture and Life Sciences, Kyungpook National University, Daegu, Republic of Korea

## Abstract

In March of 2014, a variant of novel porcine epidemic diarrhea virus (PEDV) was first identified in South Korea and found to be most closely related to the U.S. variant strain OH851. The complete genome of the KOR/KNU-1406/2014 strain was sequenced and analyzed to investigate the U.S.-strain-like variant circulating in South Korea.

## GENOME ANNOUNCEMENT

Porcine epidemic diarrhea virus (PEDV), a member of the *Alphacoronavirus* genus within the family *Coronaviridae* of the order *Nidovirales*, is a highly epizootic deadly virus with high mortality in neonatal piglets (1–3). Beginning in late 2013, large-scale outbreaks of PEDV have recurred and swept rapidly across the country, including Jeju Island. Genetic and phylogenetic analyses revealed that recent PEDV isolates in South Korea were most highly related to emergent PEDVs disseminating in the United States (4, 5). In March of 2014, pigs on a farm located in Kyungpook Province suffered from PED-like symptoms, and fecal samples from pigs with diarrhea were submitted to our laboratory. According to RT-PCR using a TGE/PED detection kit (iNtRON Biotechnology, Seongnam, South Korea), all stool samples were found to be positive for PEDV. Subsequently, the complete sequences of spike (S) protein sequences of KNU-1406 isolates from the same farm were initially determined. Nucleotide sequencing analysis showed that all four KNU-1406 strains are genetically nearly identical to each other ranging from 99.9 to 100% nucleotide (nt) homology but show 95.5 to 96.5% nt identity to PEDVs recently emerging in South Korea and the United States. However, on the basis of BLAST searches, KNU-1406 isolates exhibited 99.8 to 99.9% nt identity to a new variant strain OH851 from the United States and had the same genetic signatures found in the OH851 S protein (6), indicating that the KNU-1406 strain is a U.S.-strain-like variant of PEDV. To determine the genetic relationship of the novel Korean strain and other global PEDV strains, the complete genome of PEDV KNU-1406 was sequenced and analyzed.

The 5′ and 3′ ends of the genome of KNU-1406 were determined by rapid amplification of cDNA ends (RACE), as described previously (7). Ten overlapping cDNA fragments were generated to encompass the entire genome, pooled in equimolar amounts, and subjected to next-generation sequencing (NGS) using the Ion Torrent Personal Genome Machine (PGM) Sequencer System (Life Technologies, Carlsbad, CA) and an Ion 316 sequencing chip version 2 (Life Technologies). The KNU-1406 NGS reads were assembled using complete PEDV reference genomes from GenBank (8–12). The full-length genomic sequence of KNU-1406 is 28,029 nt in length, excluding the 3′ ploy(A) tail. The complete PEDV genome of KNU-1406 has a nucleotide identity of 96.6 to 99.7% with other complete PEDV genomes available in GenBank, with the highest nucleotide identity (99.7%) with a new variant U.S. strain, OH851 (GenBank accession number KJ399978). Comparing the complete genome of KNU-1406 to that of the U.S. strain OH851, KNU-1406 had 57 different nt, of which 1 are in the 5′ UTR, 45 in the ORF1, 4 in the S gene, 5 in ORF3, 1 in the M gene, and 1 in the N gene.

To our knowledge, this is the first time that the complete genome sequence of the new variant Korean strain of PEDV was determined. Our sequence data will provide further insights into understanding the epidemiology and evolution of PEDV and facilitating investigations on the genetic diversity of PEDV field strains in South Korea.

### Nucleotide sequence accession number.

These KOR/KNU-1406/2014 PEDV sequence data have been deposited in GenBank under the accession number KM403155.
